# Spin-orbital coupling and slow phonon effects enabled persistent photoluminescence in organic crystal under isomer doping

**DOI:** 10.1038/s41467-021-23791-9

**Published:** 2021-06-09

**Authors:** Yixuan Dou, Catherine Demangeat, Miaosheng Wang, Hengxing Xu, Bogdan Dryzhakov, Eunkyoung Kim, Tangui Le Bahers, Kwang-Sup Lee, André-Jean Attias, Bin Hu

**Affiliations:** 1 Department of Materials Science and Engineering, University of Tennessee, Knoxville, TN USA; 2 Building Blocks for FUture Electronics Laboratory, IRL 2002, CNRS - Sorbonne Université -Yonsei University, Seoul, South Korea; 3 Department of Chemical and Biomolecular Engineering, Yonsei University, 50 Yonsei-ro, Seodaemun-gu, Seoul, South Korea; 4 Univ. Lyon, ENS de Lyon, CNRS UMR 5182, Université Claude Bernard Lyon 1, Laboratoire de Chimie, Lyon, France; 5 Department of Advanced Materials and Chemical Engineering, Hannam University, Daejeon, Republic of Korea

**Keywords:** Synthetic chemistry methodology, Electronic devices

## Abstract

When periodically packing the intramolecular donor-acceptor structures to form ferroelectric-like lattice identified by second harmonic generation, our CD49 molecular crystal shows long-wavelength persistent photoluminescence peaked at 542 nm with the lifetime of 0.43 s, in addition to the short-wavelength prompt photoluminescence peaked at 363 nm with the lifetime of 0.45 ns. Interestingly, the long-wavelength persistent photoluminescence demonstrates magnetic field effects, showing as crystalline intermolecular charge-transfer excitons with singlet spin characteristics formed within ferroelectric-like lattice based on internal minority/majority carrier-balancing mechanism activated by isomer doping effects towards increasing electron-hole pairing probability. Our photoinduced Raman spectroscopy reveals the unusual slow relaxation of photoexcited lattice vibrations, indicating slow phonon effects occurring in ferroelectric-like lattice. Here, we show that crystalline intermolecular charge-transfer excitons are interacted with ferroelectric-like lattice, leading to exciton-lattice coupling within periodically packed intramolecular donor-acceptor structures to evolve ultralong-lived crystalline light-emitting states through slow phonon effects in ferroelectric light-emitting organic crystal.

## Abstract (web summary)

Understanding the underlying spin- and photo-physics of ultralong-lived light emission in organic materials remains a significant challenge. Here, the authors report spin-orbital coupling and slow phonon effects in light-emitting organic molecules and elucidate their underlying physics.

## Introduction

The recent experimental studies have shown an emerging phenomenon, where a persistent light emission can be observed with the photoluminescence (PL) lasting from seconds to minutes and hours after ceasing photoexcitation, leading to an afterglow phenomenon in organic molecules at room temperature^1–8^. The persistent light emission in organic materials indicate new fundamental mechanisms that can evolve optically generated excitons with a lifetime normally less than microseconds into ultralong-lived light-emitting states, with the lifetime from seconds to minutes and hours at room temperature. Notably, the triplets have been widely considered to be responsible for persistent light emission in organic molecules. In this scenario, it has been proposed that the triplets can directly recombine into phosphorescence or convert into singlets through the intersystem crossing, leading to persistent PL based on phosphorescence and delayed fluorescence. It should be noted that both phosphorescence and delayed fluorescence require a substantial spin–orbital coupling (SOC) to flip the spins of triplets toward either direct recombination or intersystem crossing^9,10^. Therefore, establishing a SOC becomes a critical issue to generate phosphorescence and delayed fluorescence toward realizing a persistent PL. It has been considered that directed heavy atom effect^10,11^, aromatic aldehyde^12^, and deuterated carbon structures^13,14^ can generate a SOC in organic molecules to flip the spins. Recently, by using magnetic field effects of PL, we have observed that the charge-transfer states formed between donor and acceptor components within light-emitting exciplex systems can directly establish a SOC by asymmetrically polarizing the orbital wavefunctions from optically generated dipoles^15,16^. However, the persistent PL is still lacking the direct evidence on SOC to experimentally verify the role of triplets through either phosphorescence or delayed fluorescence. Nonetheless, it demands much fundamental effort to understand the underlying mechanisms that evolve the light-emitting states into ultralong-lived states to generate a persistent PL. Interestingly, it has been reported that the isomer molecules function as donor to induce the charge-separated states with intrinsic molecules, and consequently generates a persistent PL based on proposed phosphorescence from triplets in carbazole derivatives^17^. This observation indicates that inducing charge transfer plays an important role in developing ultralong-lived light-emitting states in organic molecules. On the other hand, it was reported in the 1970s that the singlet excitons associated with photoinduced molecular structural twist can give rise to a persistent PL with the lifetime in the order of seconds in *p*-(9-anthryl)-*n*, *n*-dimethylaniline derivatives at room temperature^18–20^.

In this work, we utilized magnetic field effects and photoexcitation-assisted Raman spectroscopy to explore SOC and slow phonon effects to understand the underlying spin-physics and photophysics of evolving photoexcited excitons into ultralong-lived light-emitting states in developing persistent light emission based on our synthesized light-emitting CD49 molecules (9-(3-(5-bromopyridin-3-yl)prop-2-yn-1-yl)-9H-carbazole), with the PL lifetime in the order of seconds. The long-wavelength persistent PL peaked at 542 nm observed from the CD49 molecular crystal demonstrates magnetic field effects, showing as crystalline charge-transfer excitons with singlet spin characteristics through the formation of electron–hole pairs within Coulomb capture radius in periodically packed intramolecular donor–acceptor (D–A) structures. Essentially, the periodically packed intramolecular D–A structures act as ferroelectric-like dipolar lattice, shown by strong second-harmonic generation (SHG) signal, to host crystalline intermolecular charge-transfer excitons toward establishing exciton–lattice coupling, providing a ready condition for realizing persistent PL in the CD49 molecular crystal. Furthermore, our photoinduced Raman spectroscopy revealed an unusual slow relaxation of photoexcited lattice vibrations in the order of seconds upon applying/removing photoexcitation in the CD49 molecular crystal. This unusual slow relaxation of photoexcited lattice vibrations provides a basic mechanism to evolve crystalline charge-transfer excitons into ultralong-lived light-emitting states through exciton–lattice interaction within periodically packed intramolecular D–A structures with ferroelectric-like dipolar polarization toward realizing persistent PL. On the other hand, the CD49 molecular crystal functions as a common p-type semiconductor where photoexcited electrons and holes have much shorter and longer lifetimes shown by distinctly low and high mobilities, serving as minority and majority carriers, respectively. This can largely decrease electron–hole pairing probability within Coulomb capture radius due to unbalanced electron/hole carriers, causing difficulty to establish crystalline intermolecular charge-transfer excitons for realizing a persistent PL at room temperature. Consequently, it requires n-type doping to balance minority/majority carriers to increase the electron–hole pairing probability within Coulomb capture radius toward forming crystalline intermolecular charge-transfer excitons within organic molecular crystals. Our magneto-dielectric studies show that crystalline intermolecular charge-transfer excitons are coupled with ferroelectric-like lattice, leading to exciton–lattice coupling in periodically packed intramolecular D–A structures. Based on this exciton–lattice coupling, the slow phonon effects provide a fundamental possibility to evolve crystalline intermolecular charge-transfer excitons into ultralong-lived light-emitting states toward developing persistent PL, once the carrier-balancing mechanism is activated in organic molecular crystal.

## Results

### Materials characterizations

**Fig. 1:**
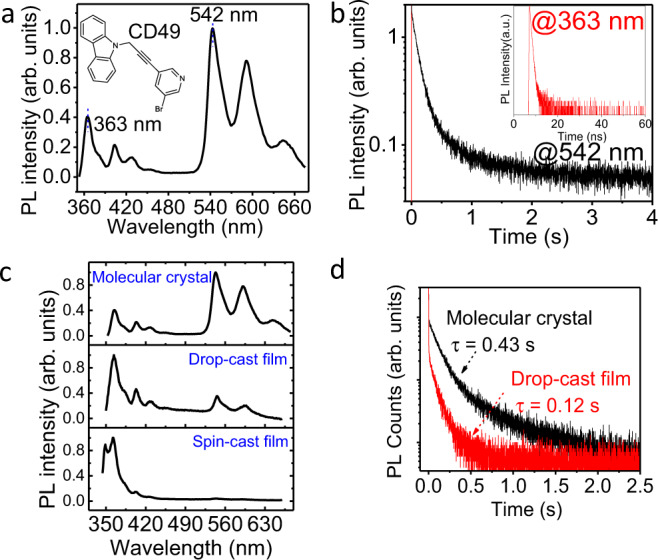
PL characteristics for crystal, drop-cast film, and spin-cast film (CD49). **a** PL spectra measured from CD49 molecular crystal under 325 nm laser excitation (Inset: chemical structure for CD49). **b** PL decay dynamics for short-wavelength PL peaked at 363 nm and long-wavelength PL peaked at 542 nm. PL lifetime was fitted with exponential decay equation, the average lifetime was calculated by the equation: ${\tau }_{{\mathrm{ave}}}=\frac{\mathop{\sum }\nolimits_{i=1}^{n}{\alpha }_{n}\times {\tau }_{n}^{2}}{\mathop{\sum }\nolimits_{i=1}^{n}{\alpha }_{n}\times {\tau }_{n}}$. **c** PL spectra for CD49 molecular crystal, drop-cast film, and spin-cast film. **d** PL decay dynamics for CD49 molecular crystal and drop-cast film.

The light-emitting CD49 molecular crystal shows short-wavelength and long-wavelength PL peaked at 363 and 542 nm with short and ultralong lifetimes at room temperature, known as prompt and persistent light emission (Fig. 1a, b). The long-wavelength PL peaked at 542 nm leads to an afterglow phenomenon lasting up to 2 s after ceasing photoexcitation (Supplementary Fig. 1). The curve-fitting on the time-resolved PL decay spectrum shows a lifetime of 0.43 s, indicating ultralong-lived light-emitting states formed in the CD49 molecular crystal at room temperature. In contrast, the short-wavelength prompt PL peaked at 363 nm exhibits a lifetime of 0.45 ns, representing short-lived intramolecular excitons, known as Frenkel excitons. Interestingly, the persistent PL is dependent on crystallinity, as shown by the large decrease of persistent PL intensity from molecular crystal to drop-cast film, and disappeared in amorphous spin-cast film, as illustrated in Fig. 1c. Simultaneously, the lifetime of long-wavelength persistent PL is decreased from 0.43 to 0.12 s when lowering the crystallinity from molecular crystal to drop-cast film (Fig. 1d). The different morphological structures in molecular crystal, drop-cast film, and spin-cast film are reflected by optical images shown in Supplementary Fig. 2. The crystallinity-dependent persistent PL indicates that the ultralong-lived light-emitting states are essentially crystalline intermolecular charge-transfer excitons formed within periodically packed intramolecular D–A structures in the CD49 crystal.

### Crystalline intermolecular charge-transfer excitons

**Fig. 2:**
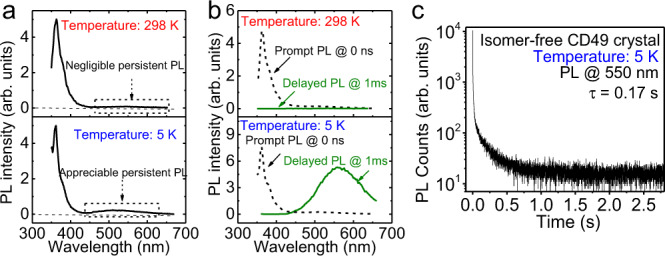
PL characteristics in isomer-free CD49 crystal at room temperature (298 K) and low temperature (5 K). **a** Steady-state PL spectra. **b** PL spectra at different delay times at 0 ns and 1 ms at 298 K and 5 K. **c** PL lifetime at 550 nm under 5 K.

It should be emphasized that organic materials especially carbazole derivatives are well-known as p-type semiconductors, where photoexcited electrons and holes possess much unbalanced lifetimes due to distinctly low and high mobilities^21,22^, acting as minority and majority carriers. The distinctly unbalanced carrier lifetimes can largely lower the electron–hole pairing probability within Coulomb capture radius and causes difficulty to generate crystalline intermolecular charge-transfer excitons in the organic molecular crystal at room temperature. It has been found that introducing isomer molecules in carbazole derivatives can induce persistent PL, and lacking the isomer leads to the disappearance of persistent PL^17^. It was proposed that the isomer, namely benzoindole (Bd), can form charge transfer with intrinsic carbazole derivatives toward developing the persistent PL. Here, we consider that the isomer molecules are essentially functioning as n-type dopants to donate electrons into the conduction band of CD49 molecular crystal to provide a carrier-balancing mechanism toward forming crystalline charge-transfer excitons through electron–hole pairing action within Coulomb capture radius in periodically packed intramolecular D–A structures. Indeed, our CD49 samples prepared with the commercial carbazole (Sigma) contain the isomer molecules (CD102) as demonstrated by the high-performance liquid chromatography (HPLC) data shown in Supplementary Fig. 3. When synthesizing the CD49 target from pure carbazole, the long-wavelength persistent PL becomes disappeared in the isomer-free CD49 crystal at room temperature (Fig. 2a). Interestingly, the persistent PL can be recovered by lowering the temperature in the absence of isomer molecules (Fig. 2b). At 5 K, the long-wavelength PL shows the ultralong lifetime of 0.17 s in the isomer-free CD49 crystal. This experimental observation provides the following three critical understandings. First, the crystalline intermolecular charge-transfer excitons are essentially formed between periodically packed intramolecular D–A structures, rather than between isomer and CD49 molecules, to develop a persistent PL. Second, the isomer molecules are functioning as n-type dopants to provide a carrier-balancing mechanism to increase electron–hole pairing probability toward forming crystalline intermolecular charge-transfer excitons within Coulomb capture radius. Third, when the n-type doping molecule (CD102) is absent, lowering the temperature can increase the Coulomb capture radius, increasing the electron–hole pairing probability to form crystalline intermolecular charge-transfer excitons in organic crystal. It is also noted that isomer-free and isomer-containing CD49 crystals show slightly different long-wavelength persistent PL spectra, but similar short-wavelength prompt PL. This indicates that the isomer molecules can somewhat change the crystalline packing in CD49 molecular crystal.

**Fig. 3:**
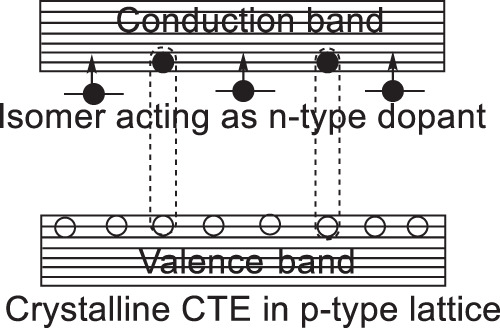
Schematic diagram to show the formation of electron–hole pairs in crystalline lattice through Coulomb capture between minority electrons and majority holes toward generating crystalline charge-transfer excitons (CTE) in p-type organic crystal under n-type doping. Isomer molecules are functioning as n-type dopants to donate electrons into p-type organic crystal.

Now, we discuss the formation of crystalline intermolecular charge-transfer excitons within the periodically packed intramolecular D–A structures in the CD49 molecular crystal. When photoexcitation generates equal numbers of electrons and holes, the largely different carrier lifetimes can inevitably decrease electron–hole pairing probability within Coulomb capture radius, presenting a difficulty to form crystalline intermolecular charge-transfer excitons for realizing a persistent PL, shown by the absence of persistent PL in the isomer-free CD49 molecular crystal. However, this difficulty can be removed by introducing n-type dopants into p-type materials through a carrier-balancing mechanism. Figure 3 schematically illustrates that the isomer molecules (CD102) act as n-type dopants to activate the carrier-balancing mechanism toward increasing the electron–hole pairing probability for forming crystalline intermolecular charge-transfer excitons in the CD49 molecular crystal. Essentially, the n-type doping effects can be realized by satisfying two necessary conditions: (i) energy conservation while donating electrons from n-type dopants to the conduction band of intrinsic lattice, and (ii) the electron wavefunction overlap between dopants and intrinsic lattice to allow electron donation through Dexter transfer. Clearly, isomer molecules can conveniently satisfy these two necessary conditions through lattice-substitutional occupation, enabling n-type doping effects toward forming crystalline intermolecular charge-transfer excitons to realize a persistent PL in p-type molecular crystals.

We should point out that, without n-type doping from isomer molecules, lowering temperature can offer an alternative approach to increase the probability for minority electrons to pair with majority holes by increasing the Coulomb capture radius toward forming the crystalline intermolecular charge-transfer excitons. The Coulomb capture radius can be given by ${r}_{\mathrm{c}}=\frac{{e}^{2}}{4\pi \varepsilon {KT}}$, where *ε* is the dielectric constant, *K* is the Boltzmann constant, and *T* is the temperature. Theoretically, lowering the temperature can largely increase the Coulomb capture radius, consequently increasing the probability of finding minority electrons to form electron–hole pairs toward forming crystalline intermolecular charge-transfer excitons. We can see in Fig. 2c that lowering the temperature to 5 K can clearly induce the long-wavelength persistent PL with a lifetime of 0.17 s in the isomer-free CD49 molecular crystal. This low temperature-induced persistent PL in the isomer-free CD49 crystal verifies that the ultralong-lived light-emitting states are the crystalline intermolecular charge-transfer excitons formed between periodically packed intramolecular D–A structures toward developing persistent PL. At room temperature when artificially adding the isomer molecules (CD102) into isomer-free CD49 solution, the prepared crystal shows the recovered long-wavelength persistent PL with a lifetime of 0.17 s at room temperature (Supplementary Fig. 4). This further indicates that the isomer molecules embedded into the crystalline structure function as n-type dopants to provide the carrier-balancing mechanism necessary to increase electron–hole pairing probability within Coulomb capture radius toward forming crystalline charge-transfer excitons within periodically packed intramolecular D–A structures to realize a persistent PL at room temperature.

### Spin characteristics of ultralong-lived light-emitting states

**Fig. 4:**
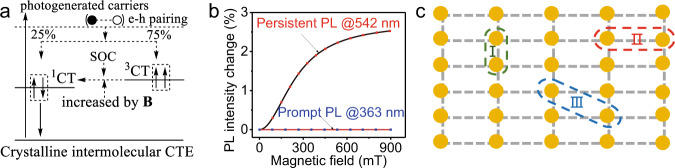
Characteristics of crystalline intermolecular charge-transfer excitons (CTE) in organic molecular crystal. **a** Diagram to show electron–hole pairing to form singlet and triplet crystalline intermolecular charge-transfer excitons (CTE) with 25% and 75%. SOC flips spins, enabling exothermic triplet-to-singlet intersystem crossing in crystalline intermolecular charge-transfer excitons. Magnetic field increases triplet-to-singlet intersystem crossing by introducing faster and slower spin precessions due to different *g* factors on electrons and holes. **b** Magnetic field effects measured for long-wavelength persistent PL (at 542 nm with ultralong lifetime of 0.43 s) and short-wavelength prompt PL (at 363 nm with short lifetime of 0.45 ns; *λ*_ex_ = 325 nm). **c**. The diagram shows crystalline intermolecular charge-transfer excitons through three different electron–hole captures labeled with **I**, **II**, and **III** types within crystalline lattice from periodically packed intramolecular D–A structures. Each dot represents an intramolecular D–A structure.

Now, we discuss the spin characteristics of ultralong-lived light-emitting states by using magnetic field effects of PL. It is noted that both singlet and triplet states have been proposed to be responsible for persistent PL through the optically induced molecular twist and de-trapping dynamics^18–20^. Recently, the triplets have been widely considered to discuss persistent PL in organic molecules^1,2,6,23^. It was further proposed that the triplet excitons, converted from optically generated singlet excitons through the intersystem crossing, can undergo a Dexter triplet–triplet transfer toward enhancing persistent PL based on carbazole-bromodibenzofuran molecules^24^. However, the spin characteristics of ultralong-lived light-emitting states are still experimentally lacking to support the theoretically proposed triplet states in persistent PL. Here, we explore the spin characteristics of crystalline intermolecular charge-transfer excitons by using magnetic field effects of PL. In general, magnetic field effects of PL can provide direct evidence on whether the light-emitting states are singlets or triplets, identifying the spin characteristics of light-emitting states in organic materials. We have shown that magnetic field effects of PL can be conveniently observed from intermolecular charge-transfer excitons formed between donor and acceptor components in organic molecules^15,25,26^. When photoexcited electrons and holes are randomly paired at donor and acceptor interfaces, both singlet and triplet intermolecular charge-transfer excitons are formed with the ratio of 1:3 through non-germinate recombination in organic materials, as shown in Fig. 4a. Notably, the triplets are normally having higher energy than the singlets in intermolecular charge-transfer excitons governed by spin-exchange interaction, opposite to intramolecular Frenkel excitons, where the singlets are always having higher energy than triplets due to the kinetic energy difference governed by Pauli exclusion-controlled wavefunction distributions in intramolecular states^27–29^. We should emphasize that intermolecular charge transfer can simultaneously induce electrical dipole (D^+^ → A^−^) and spin dipoles (D^+^ and A^−^) due to unpaired electrons under photoexcitation. We have shown that an electric–magnetic coupling can be formed between photoinduced electric dipoles and spin dipoles within charge-transfer states, presenting an artificially engineered SOC to flip the spins^25,26^. Essentially, this induces an intersystem crossing from high-energy triplets to low-energy singlets, an exothermic process occurring in intermolecular light-emitting states. Furthermore, we have observed that a magnetic field can increase the triplet-to-singlet intersystem crossing in intermolecular charge-transfer excitons, generating positive magnetic field effects, where the PL intensity increases with magnetic field in the case that singlets serve as light-emitting states^26,30^. Therefore, magnetic field effects of PL have been commonly observed in fluorescence^26,31^, phosphorescence^32,33^, delayed fluorescence^9^, and lasing emission^34^ in organic molecules when intermolecular-type excited states, such as charge-transfer states, exciplexes, excimers, and polaron pairs are involved in light emission, as verified by positive magnetic field effects of PL in organic mixtures, where singlets serve as light-emitting states in Supplementary Fig. 5. Figure 4b shows that magnetic field effects are clearly occurring and lacking in long-wavelength persistent PL and short-wavelength prompt PL, respectively. Here, our positive magnetic field effects provide an immediate evidence that the ultralong-lived light-emitting states possess singlet spin characteristics toward generating a persistent PL in our CD49 molecular crystal. It should be emphasized that the singlet spin characteristics of ultralong-lived light-emitting states are conserved, when artificially adding the isomer molecules (CD102) functioning as n-type dopants into isomer-free CD49 solution to form a crystal, shown by positive magnetic field effects of persistent PL in isomer-containing CD49 crystal (Supplementary Fig. 4d). Essentially, the ultralong-lived light-emitting states are evolved from crystalline intermolecular charge-transfer excitons formed between different lattice units in periodically packed intramolecular D–A structures, as illustrated in Fig. 4c.

In general, magnetic field effects can be observed at low and high fields (<10 mT and >10 mT), when hyperfine interaction and SOC govern the singlet–triplet intersystem crossing, respectively^9,35,36^. The early studies in the 1970s showed that the delayed fluorescence in aromatic structures can exhibit magnetic field effects at the lower field (<10 mT)^37,38^. This indicates that aromatic molecules are dominated by hyperfine interaction in developing intersystem crossing, giving rise to magnetic field effects at low field. For the persistent PL, magnetic field effects obviously occurred at the high field up to 900 mT. This provides direct evidence that the ultralong-lived light-emitting states are formed with a SOC within periodically packed intramolecular D–A structures. When SOC is established, there is a question on whether the persistent PL involves thermally activated delayed fluorescence (TADF). The TADF is an endothermic process to convert triplet excitons into singlet excitons, extending fluorescence lifetime from nanoseconds to microseconds to generate a delayed fluorescence^39,40^. In TADF, SOC provides a necessary spin-flipping mechanism to convert non-emissive triplet excitons into light-emitting singlet excitons at the condition that the singlet–triplet energy difference is matched by thermal energy. We should note that the singlet–triplet intersystem crossing can often occur in electron–hole pairs, such as charge-transfer states and polaron pairs commonly observed in organic OLEDs^41–43^. However, the singlet–triplet intersystem crossing occurring in electron–hole pairs does not appreciably extend the fluorescence lifetime, which is fundamentally different from TADF phenomenon. Nonetheless, whether the TADF is involved in the persistent PL is an important issue. We should note that the TADF lifetime can be appreciably decreased by increasing temperature because the thermal energy facilitates the triplet-to-singlet conversion^44,45^. However, the persistent PL lifetime exhibits a slight increase in the order of seconds from 0.35 to 0.43 s, when the temperature is increased from 5 to 295 K (Supplementary Fig. 6), opposite to the TADF phenomenon. Therefore, the persistent PL is not governed by the TADF.

It has been reported that crystalline packing can induce a long-wavelength PL with the ultralong lifetime of 5.4 ms in the organic crystal [2,5-dihexyloxy-4-bromobenzaldehyde] at room temperature^10^. This long-wavelength PL with an ultralong lifetime was proposed to be a phosphorescence. This leads to the hypothesis that SOC can be formed to activate the T → S_0_ transition with phosphorescence outcome in crystalline molecular packing. However, direct experimental evidence on crystalline packing-induced SOC is still lacking. We have shown that SOC can be generated once the charge-transfer states are formed in organic D–A structures in the absence of heavy elements in organic molecules^15,16^. Essentially, charge-transfer states simultaneously possess electrical and spin polarizations with mutual interaction, leading to an electric–magnetic coupling shown as an artificially engineered SOC. Normally, charge-transfer states are formed between different molecules with high and low electron negativities. However, charge-transfer states can still be formed between the same type of molecules, exampled as light-emitting excimers occurring between carbazole units in poly(*N*-vinyl carbazole)^46,47^. In this case, the same type of molecules can exhibit different electron negativities caused by inhomogeneous structural morphologies, leading to partial charge transfer. Here, our magnetic field effects occur in persistent PL through crystalline intermolecular charge-transfer excitons. Essentially, crystalline intermolecular charge-transfer excitons are generated through the formation of electron–hole pairs within Coulomb capture radius in periodically packed intramolecular D–A structures, when photogenerated carriers occurred in organic molecular crystals. When the photoexcited electrons and holes are randomly paired within crystalline lattice, both singlet and triplet crystalline intermolecular charge-transfer excitons are formed with the ratio of 1:3 based on spin statistics, as indicated in Fig. 4a. When the spin-flipping mechanism is activated by SOC, the triplets can be spin-flipped into singlets through an exothermic process, leading to triplet-to-singlet intersystem crossing in intermolecular charge-transfer excitons. Particularly, an external magnetic field can enhance the spin-flipping mechanism by introducing different spin precessions on electron and hole within an intermolecular charge-transfer exciton through different *g* factors between electron and hole, increasing the exothermic triplet-to-singlet intersystem crossing and leading to an increase in PL intensity from singlets. This reflects as positive magnetic field effects of PL, when singlets are responsible for light emission. It should be emphasized that fluorescence and phosphorescence are normally having lifetimes in the orders of nanoseconds and microseconds in organic materials, respectively. Therefore, there must be a new mechanism to evolve crystalline intermolecular charge-transfer excitons into ultralong-lived light-emitting states toward the realization of persistent PL in organic molecular crystals.

### Slow phonon effects

**Fig. 5:**
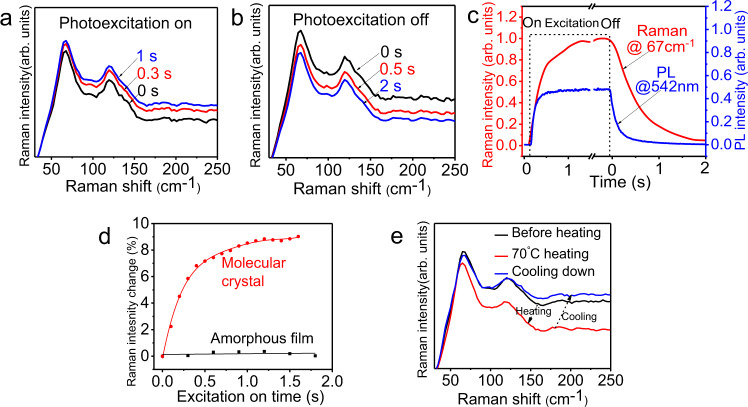
Photoexcitation-assisted Raman characterizations upon applying/removing 375 nm excitation (785 nm laser beam for Raman). **a** Raman spectra measured at different illumination times after applying 375 nm laser excitation. **b** Raman spectra measured at different times after removing 375 nm laser excitation. **c** Raman signal intensity at 67 cm^−1^ and PL intensity at 542 nm plotted as a function of time upon applying/removing 375 nm laser excitation. **d** Raman signal intensity of molecular crystal at 67 cm^−1^ and amorphous film at 59 cm^−1^ plotted as a function of time upon applying/removing 375 nm laser excitation. **e**. Raman spectra measured with heating to 70 °C and cooling down to room temperature conditions.

Now, we address the fundamental mechanism to slowly relax the crystalline intermolecular charge-transfer excitons into ultralong-lived light-emitting states toward developing a persistent PL in CD49 crystal. The crystallinity-dependent persistent PL indicates that the formation of ultralong-lived light-emitting states requires periodic packing of intramolecular D–A structures in the CD49 molecules. Amorphously packing intramolecular D–A structures does not form the ultralong-lived light-emitting states, as shown by the absence of persistent PL in spin-cast film at room temperature (Fig. 1c). We should note that the CD49 molecules carry the aggregation-induced emission (AIE) properties operated by the suppressed molecular rotations with reduced nonradiative emission upon molecular packing, leading to an increase in PL intensity with increasing concentration, as illustrated in Supplementary Fig. 7c. However, the AIE properties do not lead to a persistent PL in the absence of periodic packing of intramolecular D–A structures, as shown by amorphous packing in the spin-cast film with disappeared persistent PL (Fig. 1c). At cryogenic temperature (77 K), the frozen CD49 liquid does not show the long-wavelength persistent PL that was observed in the molecular crystal at room temperature (Supplementary Fig. 7b). Furthermore, when the higher and lower crystallinity are selected in CD49 crystals (shown as stronger and weaker polarizations of persistent PL), the persistent PL lifetime is consequently decreased from 0.39 to 0.22 s (Supplementary Fig. 8). Clearly, the periodically packing intramolecular D–A structure presents a necessary condition to evolve the crystalline charge-transfer excitons into ultralong-lived light-emitting states. Here, we utilized photoexcitation-assisted Raman spectroscopy to explore extremely slow relaxation of photoexcited lattice vibrations to understand the underlying mechanism of evolving crystalline intermolecular charge-transfer excitons into ultralong-lived light-emitting states. In our experimental method, the lattice vibrations were monitored by Raman spectroscopy while the photoexcitation was applied and removed in the CD49 crystal. Figure 5 shows the Raman spectra detected by using the 785 nm laser beam, while simultaneously applying/removing the 375 nm laser photoexcitation. We can see an interesting phenomenon: the Raman signals between 50 and 200 cm^−1^ from the lattice vibrations are slowly increasing at 0.3 and 1.0 s after the 375 nm photoexcitation is applied (Fig. 5a). After removing the 375 nm photoexcitation, the Raman signals are slowly decreasing at 0.5 and 2.0 s (Fig. 5b). As a reference, the silicon does not show any detectable slow modulation on Raman signals upon applying/removing an external photoexcitation (Supplementary Fig. 9). Essentially, our photoexcitation-assisted Raman studies indicate that applying/removing photoexcitation slowly increases/decreases the lattice vibrations to a new equilibrium within the time scale of seconds, leading to unusual slow relaxation of photoexcited lattice vibrations within periodically packed intramolecular D–A structures in the CD49 crystal, defined as slow phonon effects. Normally, photoexcited lattice vibrations can quickly evolve into a new equilibrium via the assistance from a large number of local vibrations/rotations, leading to an increased density of phonons within femtoseconds to picoseconds in semiconductors^48,49^. Here, we should emphasize that, within the persistent light-emitting CD49 molecules which carry AIE properties, the molecular vibrations/rotations are significantly restricted by ordered intermolecular packing within crystalline lattice. Apparently, the unusual slow lattice relaxation upon applying/removing photoexcitation is caused by the limited energy dissipation of photoexcited lattice vibrations to the suppressed intramolecular vibrations in periodically packed intramolecular D–A structures due to AIE properties. This leads to a hypothesis that there exists a strong coupling between optical and acoustic phonons within periodically packed intramolecular D–A structures. Consequently, suppressing the acoustic phonons through AIE properties can slow the relaxation of optical phonons toward slowly relaxing the crystalline intermolecular charge-transfer excitons. Notably, the photoexcitation-induced slow modulation of lattice vibrations is occurring within a similar time window as compared to persistent PL, as illustrated in Fig. 5c. We should note that the Raman peak positions stay unchanged upon applying/removing photoexcitation, indicating that the lattice constants do not change in the CD49 molecules under photoexcitation. As a distinct comparison, Fig. 5e shows that heating decreases the Raman signal intensity and also shifts Raman peaks to lower frequencies. This indicates that heating simultaneously expands the lattice constants and decreases the phonon density in the CD49 crystal. This confirms that slow photoexcitation-induced modulation of lattice vibrations is not a heating effect. Clearly, the photoexcitation-induced slow modulation of lattice vibrations presents a fundamental mechanism to slowly relax crystalline intermolecular charge-transfer excitons into ultralong-lived light-emitting states, if the interaction between crystalline intermolecular charge-transfer excitons and lattice vibrations occurs within periodically packed intramolecular D–A structures in the CD49 molecular crystal. We should point out that the CD49 crystal without the isomer molecules still demonstrates similar unusual slow lattice relaxation at room temperature upon applying/removing photoexcitation, shown as slowly increasing/decreasing Raman signal intensity of lattice vibrations, when the photoexcitation is applied/removed (Supplementary Fig. 10). Clearly, in isomer-free CD49 crystal, periodically packing intramolecular D–A structures still provides the unusual slow relaxation of photoexcited lattice vibrations at room temperature, presenting a ready condition to develop ultralong-lived light-emitting states. However, when the isomer molecules are removed, the crystalline intermolecular charge-transfer excitons are difficult to form in the CD49 crystal at room temperature due to largely unbalanced lifetimes between photoexcited electrons and holes, shown as the absence of persistent PL (Fig. 2a). When the Coulomb capture radius is increased by lowering the temperature, the isomer-free CD49 crystal can recover the persistent PL at 5 K with a lifetime of 0.17 s based on the already-existed unusual slow relaxation of photoexcited lattice vibrations, as indicated in Fig. 2c.

### Coupling between ferroelectric polarization and crystalline intermolecular charge-transfer excitons

**Fig. 6:**
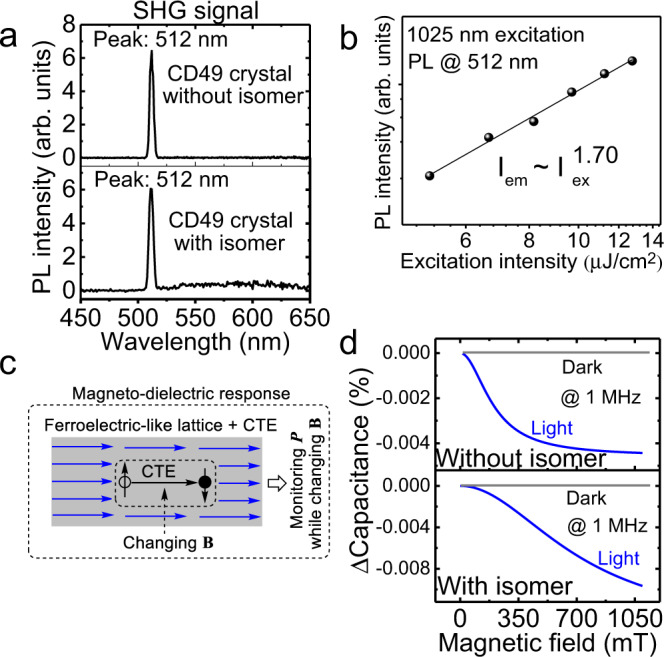
Coupling between ferroelectric polarization and crystalline intermolecular charge-transfer excitons (CTEs). **a** SHG signals for CD49 crystals prepared with and without isomer molecules under 1025 nm excitation. **b** Excitation–emission power dependence for SHG signals. **c** Principle for using magneto-dielectric response to identify coupling between ferroelectric-like lattice and crystalline intermolecular CTE. Electrical polarization ***P*** was measured at 1 MHz, while gradually increasing magnetic field **B**. When ferroelectric-like lattice is coupled with CTEs, disturbing CTEs by magnetic field leads to a change on electrical polarization, presenting magneto-dielectric response. **d** Magneto-dielectric signals were measured at 1 MHz as a function of magnetic field under 343 nm excitation as compared to dark condition.

**Fig. 7:**
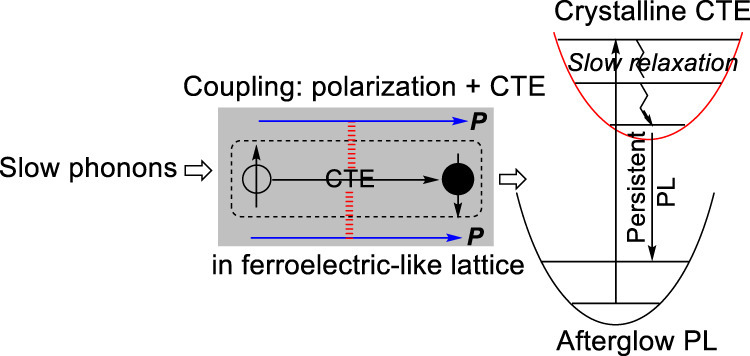
Persistent light emission is enabled by slow relaxation of photoexcited lattice vibrations when coupling between crystalline intermolecular charge-transfer excitons (CTE) and ferroelectric-like lattice is occurred. Through slow relaxation of lattice vibrations, crystalline charge-transfer excitons are slowly evolved into ultralong-lived light-emitting states, leading to a persistent PL in ferroelectric-like lattice upon periodically packing intramolecular D–A structures.

Here, we discuss the ferroelectric-like lattice in periodically packed intramolecular D–A structures in the CD49 molecular crystal based on SHG studies. The ferroelectric-like lattice can provide the necessary condition to establish crystalline exciton–lattice interaction, when crystalline intermolecular charge-transfer excitons are formed within periodically packed intramolecular D–A structures. Figure 6a shows a strong SHG for both isomer-containing and isomer-free CD49 crystals under 1025 nm laser illumination at room temperature. The SHG signals show typical quadratic power dependence with excitation intensity (Fig. 6b). The strong SHG signal confirms that the periodically packed intramolecular D–A structures are indeed formed with inverse symmetry breaking properties as ferroelectric-like lattice in both CD49 crystals prepared with and without isomer molecules. Furthermore, this SHG phenomenon implies that introducing the isomer molecules does not destroy the ferroelectric-like lattice with inverse symmetry breaking properties in the CD49 crystal. Clearly, the CD49 crystal presents as ferroelectric-like lattice by periodically packing the intramolecular D–A structures. Now, we identify whether the ferroelectric-like lattice is coupled with crystalline intermolecular charge-transfer excitons. Essentially, the coupling between the ferroelectric lattice and crystalline intermolecular charge-transfer states can establish an exciton–lattice interaction toward developing ultralong-lived light-emitting states once unusual slow relaxation of photoexcited lattice vibrations is ready. We should note that the crystalline intermolecular charge-transfer states, equivalent to aligned intermolecular charge-transfer dipoles, carry a SOC. When photogenerated electrons and holes are captured into electron–hole pairs to form intermolecular charge-transfer states, SOC can demonstrate magnetic field effects of PL (Fig. 4). Clearly, magnetic field effects represent the signature of intermolecular charge-transfer states. If the ferroelectric-like lattice is coupled with crystalline intermolecular charge-transfer states, magnetic field effects of polarization can be observed by monitoring electrical polarization with a gradually increasing magnetic field, as illustrated in Fig. 6c. Therefore, magnetic field effects of polarization present a unique method to identify the coupling between ferroelectric-like lattice and crystalline intermolecular charge-transfer states. Here, magnetic field effects of polarization were performed by monitoring the polarization signal at 1 MHz, while gradually scanning the magnetic field, at the condition that photoexcitation generates crystalline intermolecular charge-transfer dipoles. The frequency of 1 MHz was targeted at the intermolecular polarization occurring in crystalline lattice formed by periodically packing intramolecular D–A structures. Figure 6d shows magnetic field effects of polarization measured at 1 MHz for both CD49 crystals prepared with and without the isomer molecules. This observation confirms that the ferroelectric-like lattice is indeed coupled with crystalline intermolecular charge-transfer dipoles, leading to exciton–lattice coupling in periodically packing the intramolecular D–A structures. Essentially, this exciton–lattice coupling provides the ready condition to evolve crystalline intermolecular charge-transfer excitons into ultralong-lived light-emitting states through slow phonon effects within periodically packed intramolecular D–A structures. Therefore, persistent PL can be realized when crystalline intermolecular charge-transfer excitons and unusual slow relaxation of lattice vibrations are simultaneously formed within ferroelectric-like lattice from periodically packed intramolecular D–A structures, as illustrated in Fig. 7.

## Discussion

In summary, we found that the long-wavelength persistent PL is sensitive to the crystallinity in our CD49 crystal prepared with the isomer molecules. The lifetime of persistent PL is largely decreased from 0.43 to 0.12 s by lowering the crystallinity from single crystal to drop-cast film, and consequently disappeared in spin-cast film at room temperature. This indicates that crystalline intermolecular charge-transfer excitons are responsible for persistent PL upon forming electron–hole pairs through Coulomb capture within periodically packed intramolecular D–A structures. Essentially, organic molecular crystals are typical p-type semiconductors with distinctly shorter and longer lifetimes between photoexcited electrons and holes, with much lower and higher mobilities, acting as minority and majority carriers. The distinctly shorter and longer lifetimes between photoexcited electrons and holes can largely lower the electron–hole pairing probability within Coulomb capture radius, causing a difficulty to form crystalline charge-transfer excitons in p-type organic crystals. Introducing isomer molecules can enable n-type doping effect to balance the minority and majority carriers, providing a carrier-balancing mechanism to increase electron–hole pairing probability within Coulomb capture radius toward forming crystalline intermolecular charge-transfer excitons for realizing a persistent PL. Consequently, the persistent PL can be observed at room temperature in our CD49 crystal, when the isomer molecules are present, but becomes disappeared in the absence of isomer molecules due to unbalanced minority/majority carriers at room temperature. However, in the isomer-free CD49 crystal, the disappeared persistent PL can be recovered at low temperature, when the isomer molecules are absent as n-type dopants. This confirms that crystalline intermolecular charge-transfer excitons, responsible for persistent PL, are essentially formed between periodically packed intramolecular D–A structures in our CD49 molecular crystal. Interestingly, the persistent PL demonstrates positive magnetic field effects, indicating that crystalline intermolecular charge-transfer excitons carry singlet spin characteristics with spatially extended wavefunctions. To understand the basic mechanism of evolving crystalline intermolecular charge-transfer excitons into ultralong-lived light-emitting states, the slow relaxation of lattice vibrations and the exciton–lattice coupling was investigated by using photoinduced Raman spectroscopy and polarization characterizations. Our photoexcitation-assisted Raman studies found a unique phenomenon, where the Raman signals of photoexcited lattice vibrations are slowly increasing/decreasing, when applying/removing an external photoexcitation, leading to unusual slow phonon effects in the time window of seconds, providing the basic possibility to evolve intermolecular excitons into ultralong-lived light-emitting states, when the exciton-lattice coupling is occurred in crystalline lattice. Furthermore, our SHG studies indicate that periodically packed intramolecular D–A structures are formed as ferroelectric-like lattice in the CD49 crystals. Especially, the magneto-dielectric response shows that crystalline intermolecular charge-transfer excitons are coupled with the ferroelectric lattice, presenting an exciton–lattice coupling in periodically packed intramolecular D–A structures. Based on the exciton–lattice coupling, the slow relaxation of lattice vibrations can evolve crystalline intermolecular charge-transfer excitons into ultralong-lived light-emitting states, leading to a persistent PL in organic molecular crystals. Therefore, our experimental studies provide an insightful fundamental understanding of the spin-physics and photophysics of ultralong-lived light-emitting states based on SOC and slow phonon effects toward developing persistent light emission in organic crystals.

## Methods

### Synthesis

The CD49 compound was synthesized according to the following two-step procedure^50^: (1) preparation of 9-H-propargylcarbazole and (2) its subsequent Sonogashira cross-coupling with 3-bromo-5-iodopyridine.

#### 9-H-propargylcarbazole

Synthesis was adapted from the literature^51,52^. Solid KOH (1.26 g, 22.5 mmol) was added to a solution of carbazole (2.5 g, 15.0 mmol) in DMF (30 mL) at 0 °C. After stirring at this temperature for 20 min, propargyl bromide (80% solution in toluene, 2.52 mL, 22.5 mmol) was added. The reaction was allowed to stir for a further 6 h at room temperature before water (50 mL) was added. The mixture was extracted with AcOEt (3 × 30 mL) and the combined organic layer dried over CaCl_2._ The solvent was removed under reduced pressure. The crude product was purified under column chromatography using EtOAc/*n*-hexane—9/1 as eluent to obtain 9-propargylcarbazole as white crystals (2.4 g, 78%). ^1^H NMR (300 MHz, CDCl_3_) 8.08 (d, *J* = 7.8 Hz, 2H), 7.50–7.44 (m, 4H), 7.28–7.22 (m, 2H), 5.00 (s, 2H), 2.22 (t, *J* = 2.4 Hz, 1H).

#### 9-(3-(5-bromopyridin-3-yl) prop-2-yn-1-yl)-9H-carbazole

Pd(PPh_3_)_2_Cl_2_ (0.07 g, 0.1 mmol), CuI (0.04 g, 0.2 mmol), 3-bromo-5-iodopyridine (0.57 g, 2.0 mmol), 9-propargylcarbazole (0.41 g, 2.0 mmol), and triethylamine (10 mL) were added into a 100 mL two-necked flask under a nitrogen atmosphere. After stirring at room temperature for 12 h, water (15 mL) was added, and the mixture was extracted with AcOEt (3 × 15 mL). The combined organic layers were dried over CaCl_2_, and the solvents were removed under reduced pressure. The crude product was purified under column chromatography using CHCl_3_/*n*-hexane—8/2 as eluent to afford the expected product as a colorless crystal (0.65 g, 90%), mp 144–145 °C. ^1^H NMR (300 MHz, CDCl_3_): 8.57 (d, *J* *=* 2.1 Hz,1H); 8.10 (d, *J* *=* 7.8 Hz, 2H); 7,69 (dd, *J* *=* 2.1, 8.1 Hz, 1H); 7.53–7.50 (m, 4H); 7.30–7.25 (m, 2H); 7.17 (d, *J* *=* 8.1 Hz, 1H); 5.28 (s, 2H).^13^C NMR (300 MHz, CDCl_3_): 151.10, 140.77, 139.88, 138.86, 128.29, 126.03, 123.30, 120.51, 119.68, 108.80, 84.80, 82.28, 33.01.

The CD102 isomer was prepared using a similar procedure starting from pure 1H-benzo[*f*]indole. All the products were characterized by NMR with satisfactory results (Supplementary Figs. 11–16).

### Characterizations

PL in both steady and dynamic regimes were measured by the SPEX Fluorolog 3 spectrometer. The time-resolved photoexcitation source was from a pulsed laser beam (346 nm, 4 mW) generated through a harmonic generator (Ultrafast Systems LLC, third harmonic) with a Pharos laser (Light Conversion, 1 KHz, 1025 nm, 290 fs). The SHG spectrum was measured by the same excitation source as the time-resolved photoexcitation source under a frequency of 25 KHz, an 850 nm long-pass cutoff filter (Newport) was placed on the excitation beam path before the sample, an FSQ-KG5 short-pass filter (300–800 nm, Newport) was placed on the emission beam path after the sample. Photoexcitation-assisted Raman spectroscopy was characterized by using Horiba Xplora plus system confocal Raman microscope with built-in 785 nm laser beam in combination with an external photoexcitation of 375 nm laser beam. Magnetic field effects of PL (MFE-PL) were measured by monitoring PL intensity as a function of the magnetic field. The MFE amplitude is defined by the intensity change in percentage: MFE = $\frac{{{\boldsymbol{I}}}_{{{\mathbf{B}}}}-{{\boldsymbol{I}}}_{{\boldsymbol{0}}}}{{{\boldsymbol{I}}}_{{\boldsymbol{0}}}}$, where ***I***_B_ and ***I***_0_ are PL peak intensities with and without magnetic field. The low temperature was generated by the cryostat with a helium compressor (Advanced Research Systems, Inc.). Nuclear magnetic resonance (^1^H and ^13^C) spectra were obtained on a Bruker Ultra Shield 300 MHz spectrometer for CDCl_3_. The chemical shift was relative to tetramethylsilane as the internal standard. Resonance patterns are reported with the notation s (singlet), d (doublet), t (triplet), and m (multiplet). The HPLC was conducted on Agilent 1200 coupled with a DAD detector, the column is Agilent Eclipse XDB-C18 (5 µm, 4.6 × 150 mm). The HPLC injection volume of CD49 was 20 µL with 0.2 mg/mL in 70/30 acetonitrile–water (v/v).

## Supplementary information

Supplementary Information

## Data Availability

All data supporting the finding of this study are available from the authors upon reasonable request.
