# Supplementary material for: Spin-orbital coupling and slow phonon effects enabled persistent photoluminescence in organic crystal under isomer doping
Source: Nat Commun. 2021 Jun 9;12:3485. doi: 10.1038/s41467-021-23791-9 (PMC8190285; doi:10.1038/s41467-021-23791-9)
Supplement: Supplementary file 1 — Supplementary Information [file 41467_2021_23791_MOESM1_ESM.pdf]

Supplementary Information

Spin-Orbital Coupling and Slow Phonon Effects in Long Persistent Light Emission in Organic Molecules

Yixuan Dou[1], *Catherine Demangeat*[2], *Miaosheng Wang*[1], *Hengxing Xu*[1], *Bogdan Dryzhakov*[1], *Eunkyoung Kim*[2], *Tangui Le Bahers*[2], *Kwang-Sup Lee*[3], *André-Jean Attias*[2*], *Bin Hu*[1,*]

Affiliations:

[1] Department of Materials Science and Engineering, University of Tennessee, Knoxville, Tennessee, 37996, USA

[22]Building Blocks for FUture Electronics Laboratory, IRL 2002, CNRS - Sorbonne Université - Yonsei University, 50 Yonsei-ro, Seodaemun-gu, 03722 Seoul, South Korea

[3]Department of Advanced Materials and Chemical Engineering, Hannam University, Daejeon, Republic of Korea

*Corresponding authors:

André-Jean Attias: andre-jean.attias@upmc.fr

Bin Hu, E-mail: bhu@utk.edu

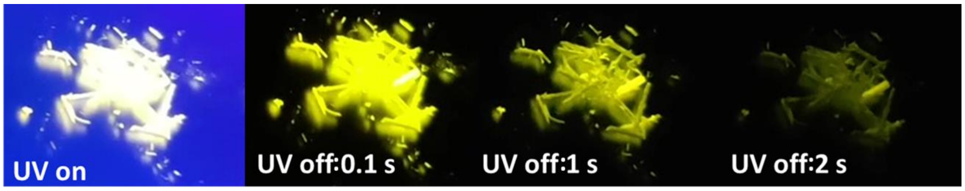

Supplementary Fig. 1. The photograph with and after UV excitation of CD49 molecular crystal

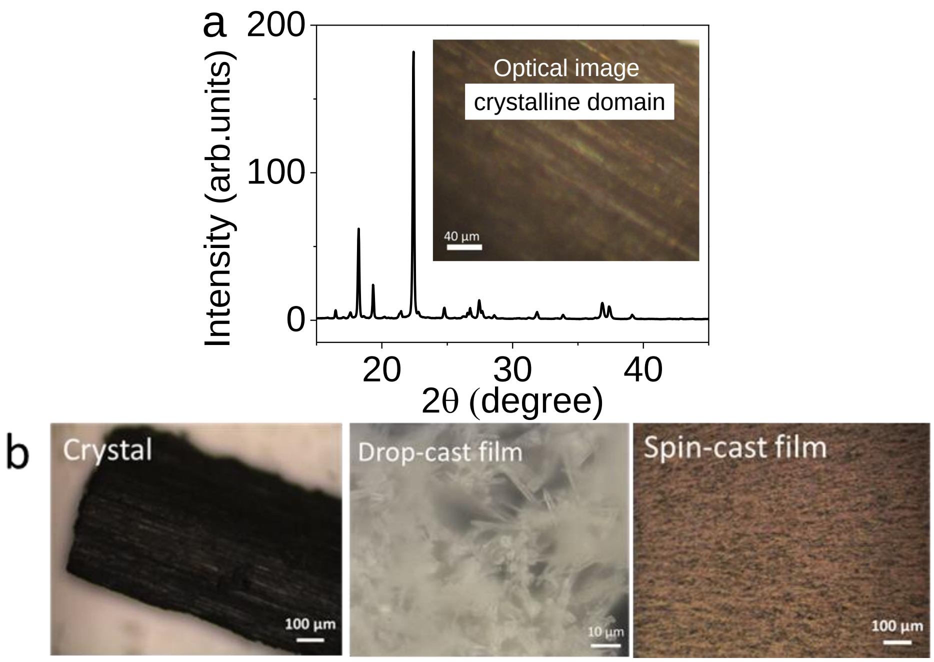

Supplementary Fig. 2. **a.** XRD pattern for CD49 molecular crystal (Inset: optical image); **b.** Optical microscopy of CD49 molecules from molecular crystal, drop-cast film, and spin-cast film.

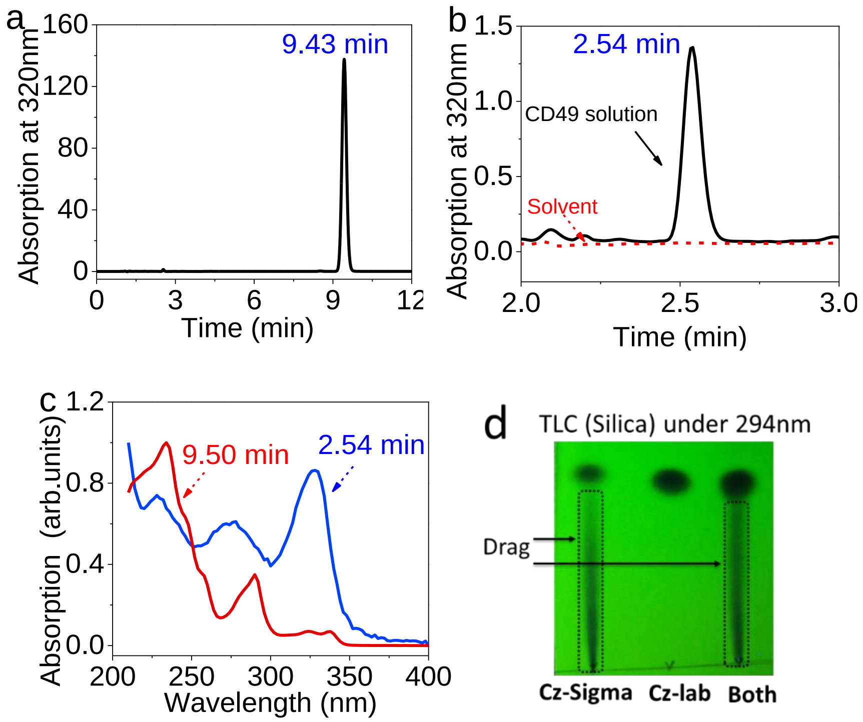

Supplementary Fig. 3. Characterization on isomer molecules in CD49 MeCN/H_2O solution. **a.** HPLC spectrum monitored at onset absorption of 320 nm of CD49 MeCN/H_2O solution; **b.** HPLC spectrum at 2.54 minutes monitored at onset absorption of 320 nm; **c.** Absorption spectra at different times; **d.** TLC results to indicate isomer impurities for CD49 samples prepared from Sigma-Cz and lab synthesized-Cz (Eluent is n-hexane/ EtOAc with the volume ratio of 9/1), and mixed Sigma-Cz and lab-synthesized Cz labeled as both. Lab-synthesized isomer-free CD49 (Cz-lab) does not show isomer impurities.

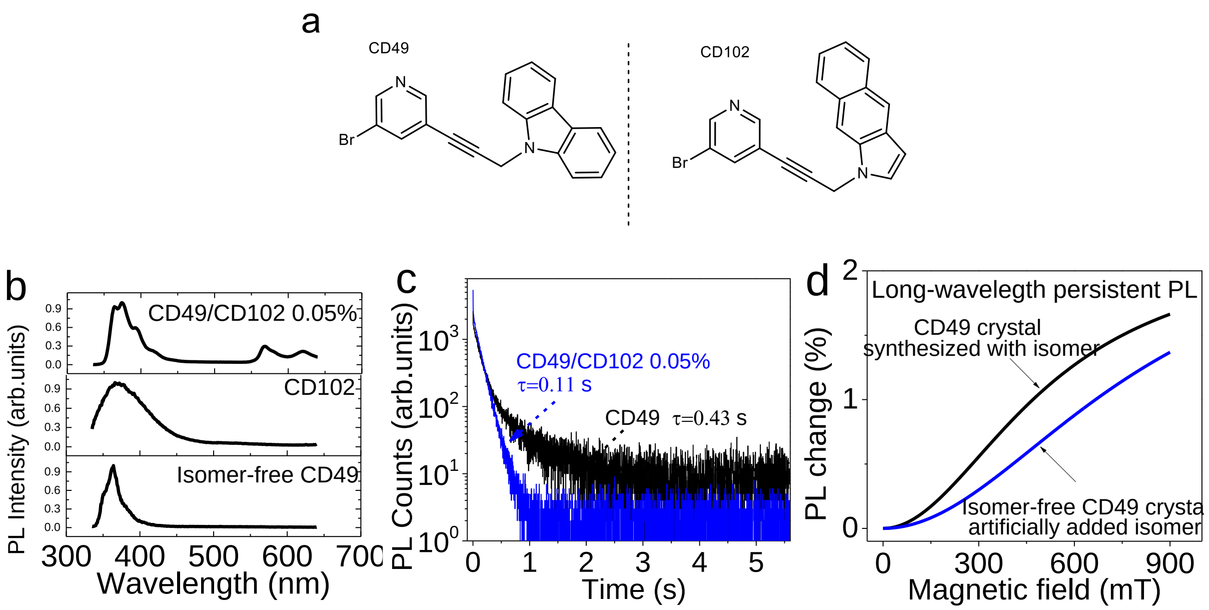

Supplementary Fig. 4. a. Chemical structures for CD49 and isomer (CD102) molecules; **b.** PL spectra of CD49 without isomer, isomer (CD102), and CD49 doped with 0.05% CD102; **c.** PL lifetimes for CD49 synthesized with isomer and isomer-free CD49 artificially added with 0.05% isomer (CD102); **d.** Magnetic field effects of persistent PL for CD49 synthesized with isomer and isomer-free CD49 artificially doped with 0.05% CD102.

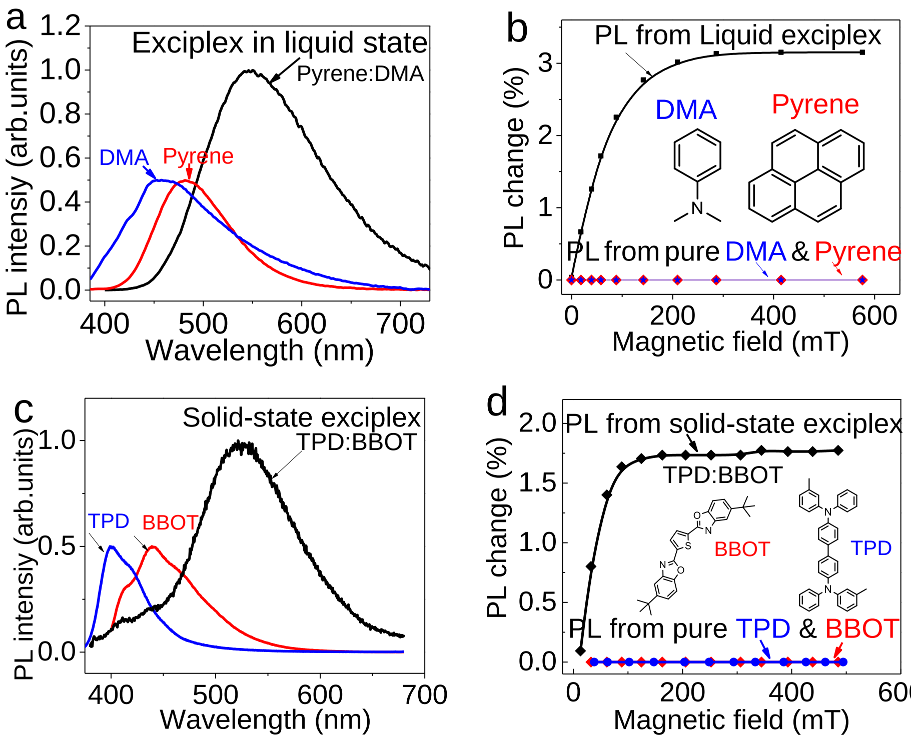

Supplementary Fig. 5. PL spectra and magnetic field effects for light-emitting exciplex systems in solid and liquid states; **a.** PL spectra for pyrene:DMA and pure pyrene/DMA solutions (DMF solvent) at 375 nm excitation. **b.** Magnetic field effects of PL; **c.** PL spectra for BBOT:TPD and pure BBOT/ TPD in solid states at 375 nm excitation; **d.** Magnetic field effects of PL.

Note: DMA, DMF, BBOT, TPF represent N,N-dimethylaniline, N,N-Dimethylmethanamide, 2,5-bis(5-tert-butyl-2-benzoxazolyl)-thiophene, and N,N'-diphenyl-N,N'-bis(3-methylphenyl)-[,11'-biphenyl]-4,4'-diamine.

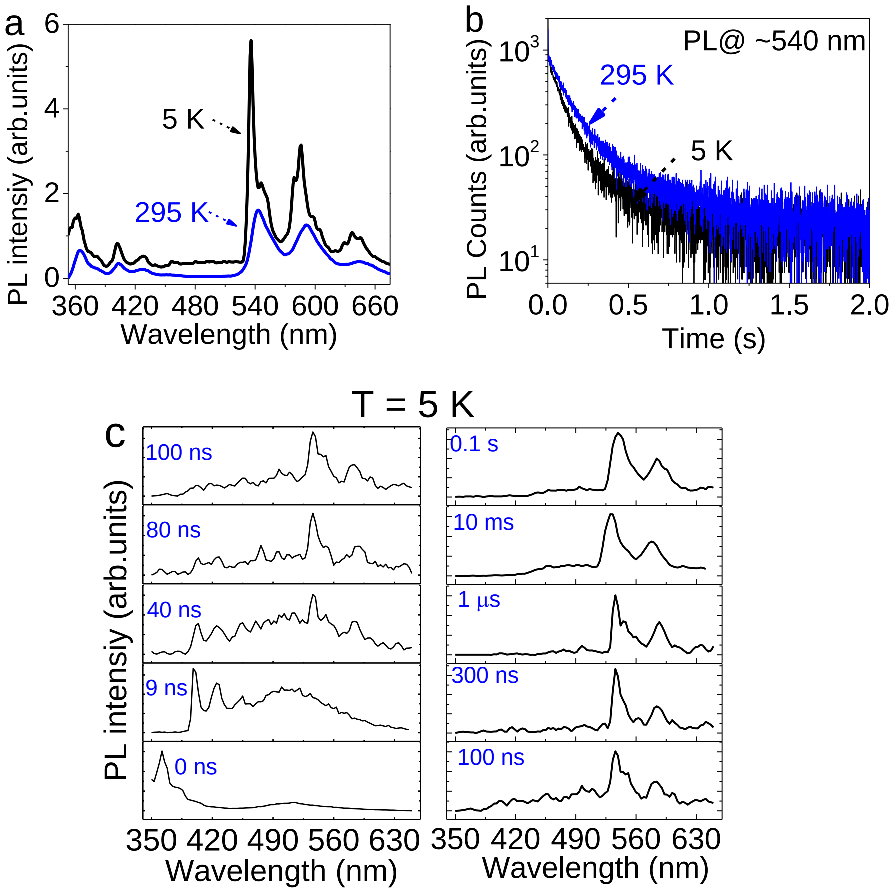

Supplementary Fig. 6. PL characteristics at different temperatures and time windows in CD49 molecular crystal synthesized with isomer molecules. **a.** Steady-state PL spectra at different temperatures; **b.** PL decay dynamics under 5 K and 295 K; **c.** Time-resolved PL spectra under 5 K.

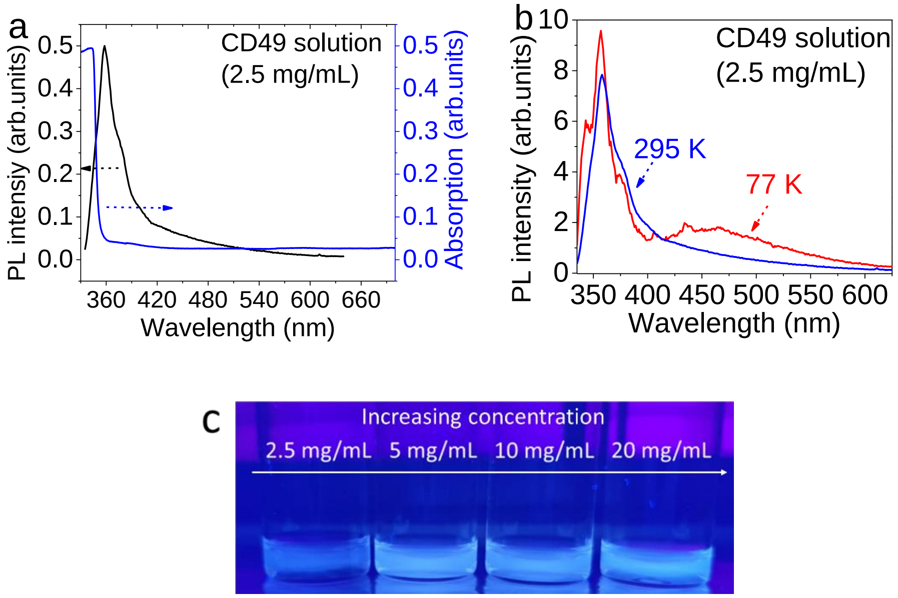

Supplementary Fig. 7. Spectral characteristics for CD49 THF solution (2.5 mg/mL); **a**. Absorption and PL spectra. **b.** PL spectra under 77 K and 295 K; **c.** Photograph with different solution concentrations.

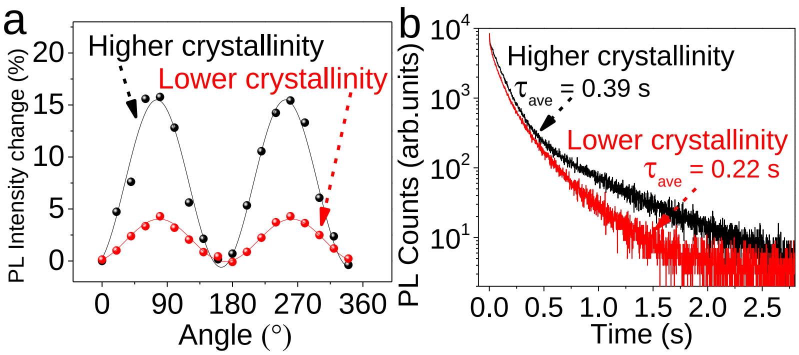

Supplementary Fig. 8. a. stronger and weaker polarized persistent PL at 542nm to show higher and lower crystallinities selected from CD49 crystals under 375 nm excitation; **b.** PL decay dynamics for higher and lower crystallinities in CD49 crystals.

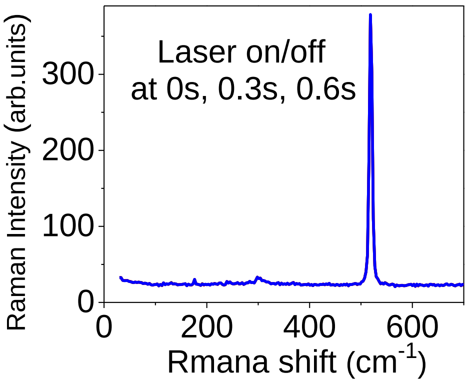

Supplementary Fig. 9. Photoexcitation-assisted Raman spectroscopy for Silicon characterized with 785 nm laser beam after applying 375 nm laser excitation. Raman signal remains unchanged upon applying/removing photoexcitation.

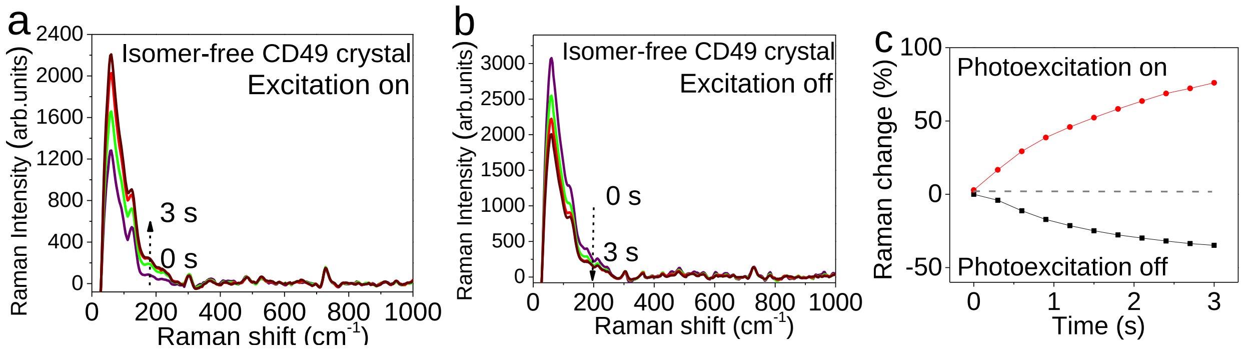

Supplementary Fig. 10. Photoexcitation-assisted Raman spectroscopy for isomer-free CD49 crystal. Raman spectroscopy was activated by using built-in 785 nm beam. Photoexcitation was applied by an external 375 nm beam. **a**. Raman spectra measured at different times after applying photoexcitation; **b**. Raman spectra measured at different times after removing photoexcitation; **c**, Raman signal intensity at 65 cm^{-1} was monitored as a function of time upon applying/removing photoexcitation.

The NMR spectra are shown for CD49 molecules in Supplementary Figure 11-16.

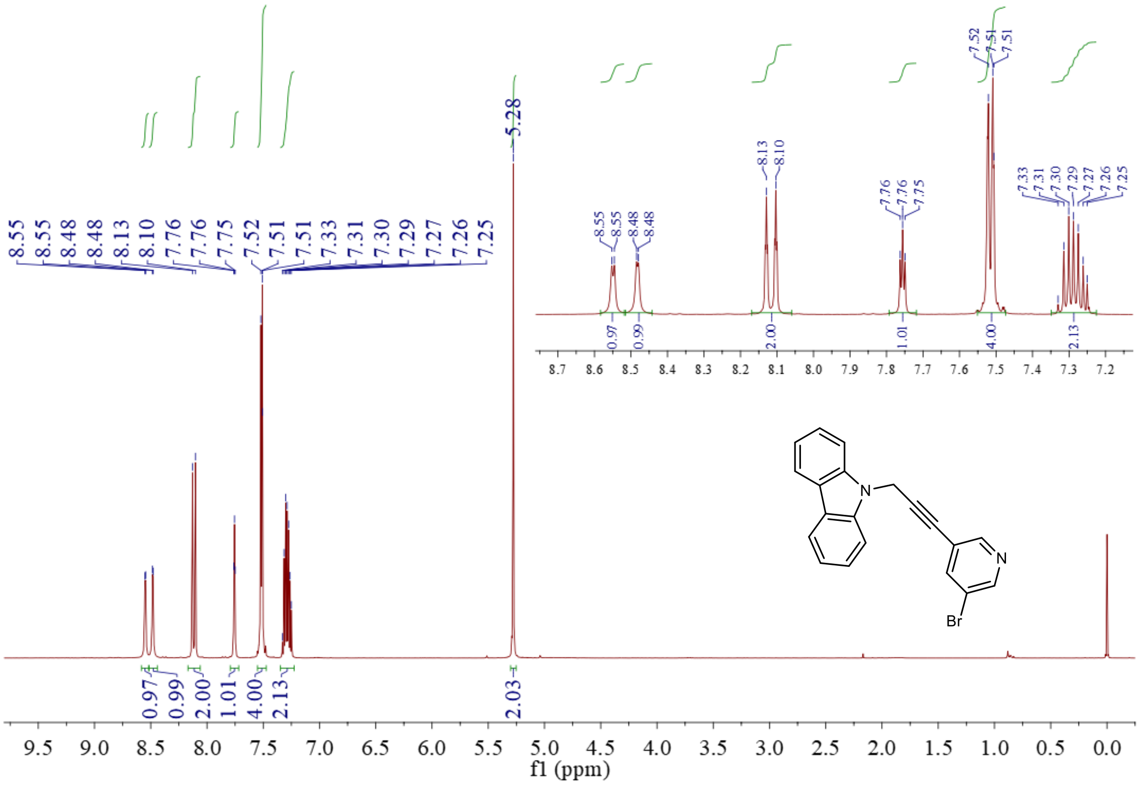

Supplementary Fig. 11. ^{1}H NMR spectrum of CD49 molecules in $CDCl_3$.

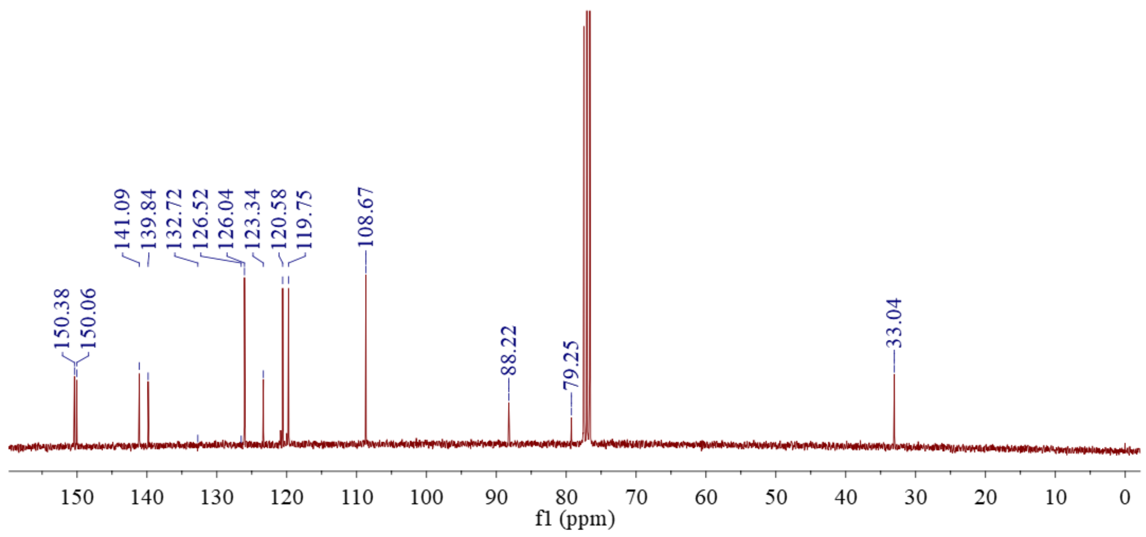

Supplementary Fig. 12: ^{13}C NMR spectrum of CD49 molecules in $CDCl_3$.

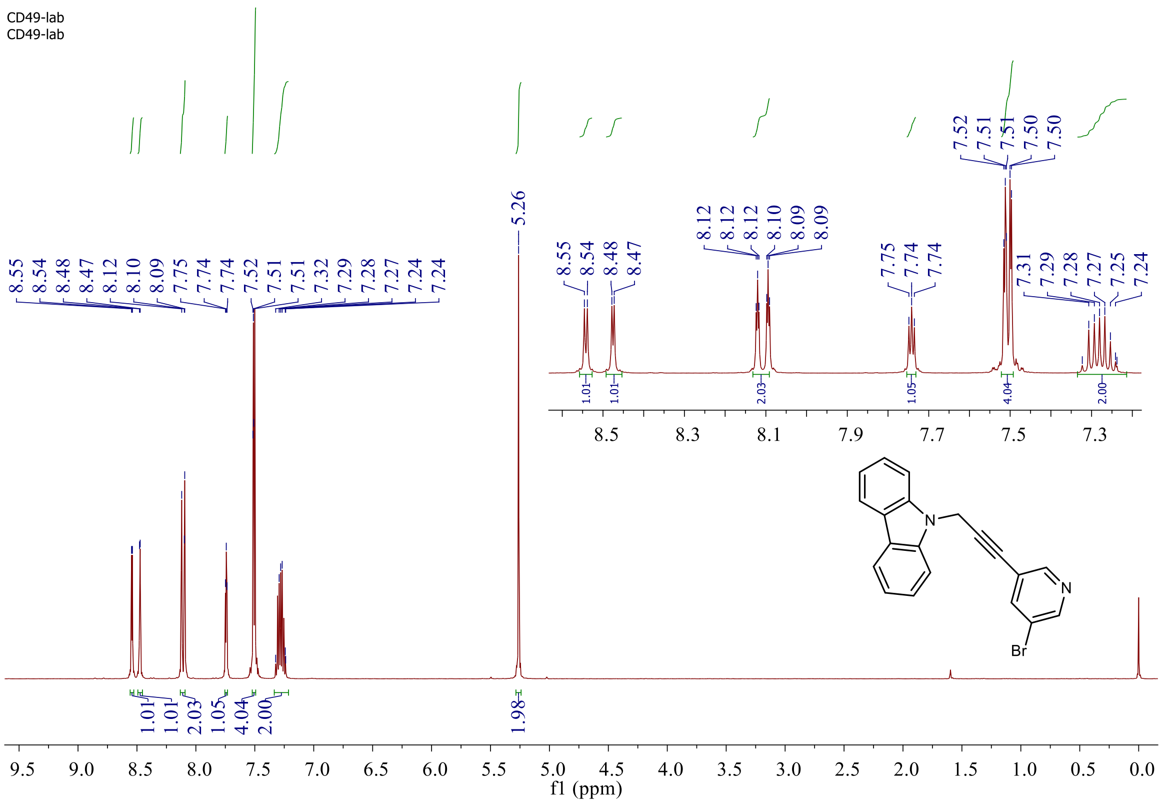

Supplementary Fig. 13: ^{1}H NMR spectrum of isomer-free CD49 in $CDCl_3$.

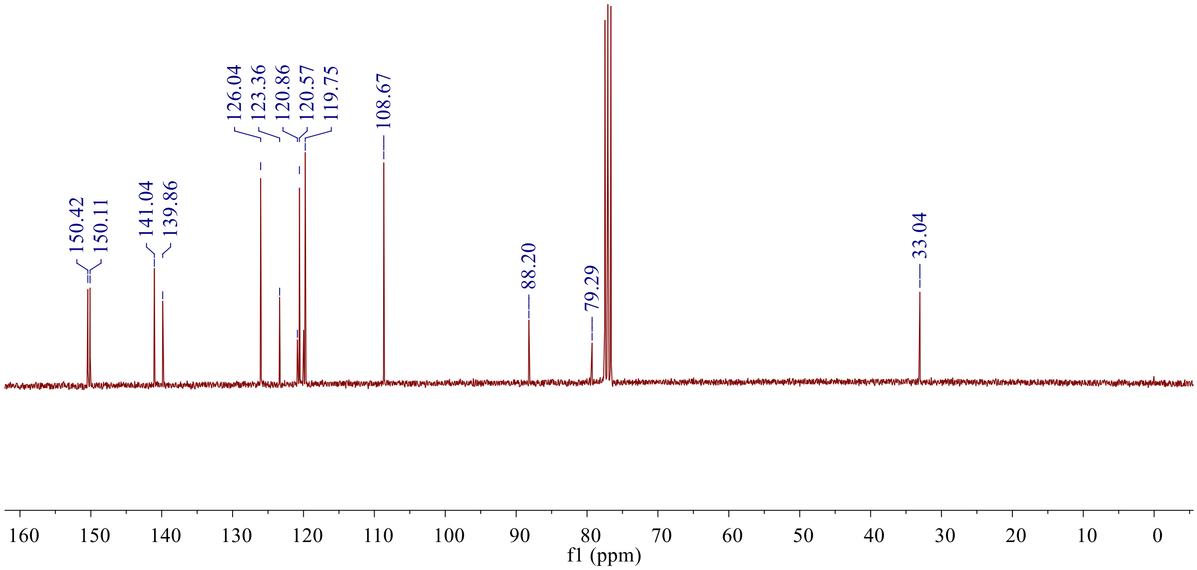

Supplementary Fig. 14: ^{13}C NMR spectrum of isomer-free CD49 in $CDCl_3$.

Isomer-free CD49: ^{1}H NMR (300 MHz, $CDCl_3$): 8.55 (d, J= 2.2 Hz,1H); 8.48 (d, J= 1.7 Hz, 1H); 8.10 (d, J= 7.8 Hz, 2H) ; 7.74 (t, J= 2.0 Hz, 1H); 7.52-7.50 (m, 4H); 7.33-7.23 (m, 2H); 5.26 (s, 2H).^{13}C NMR (300 MHz, $CDCl_3$): 150.42, 150.11, 141.04, 139.86, 126.04, 123.36, 120.57, 119.75, 108.67, 88.20, 79.29, 33.04.

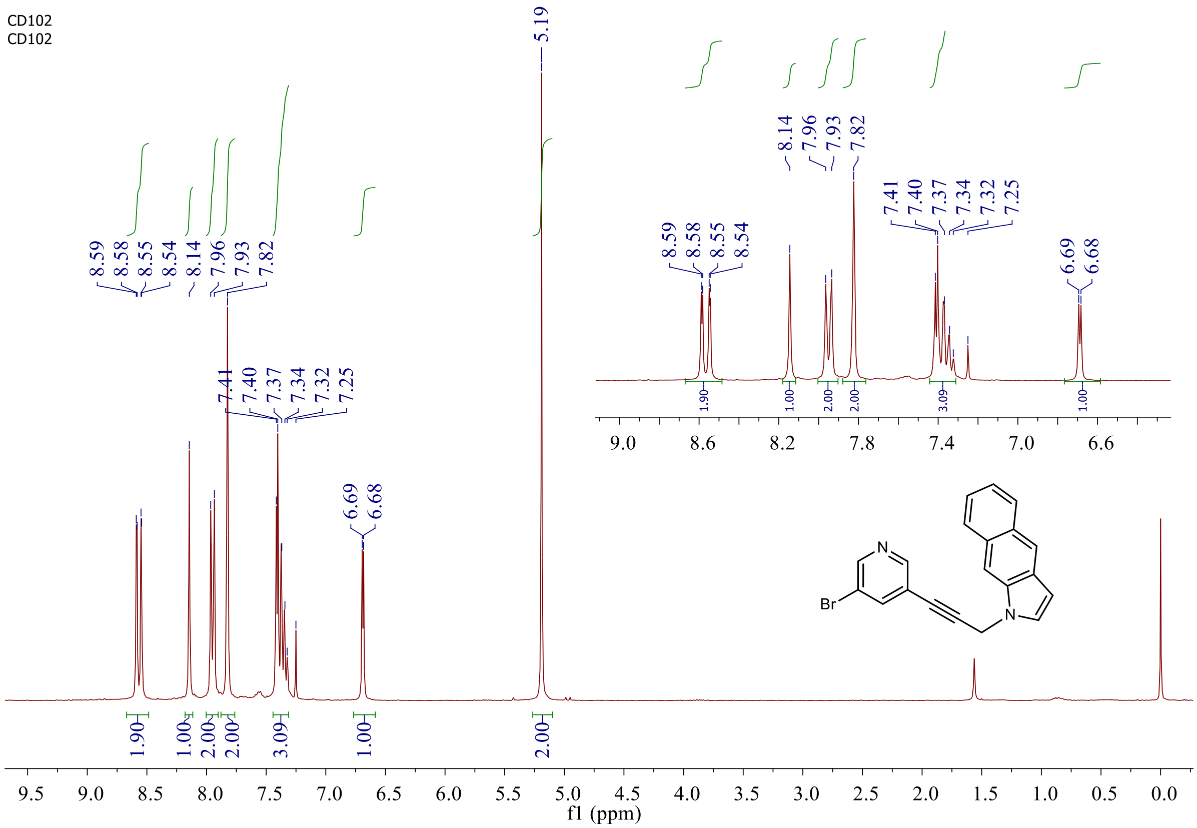

Supplementary Fig. 15: ^{1}H NMR spectrum of isomer (CD102) in $CDCl_3$.

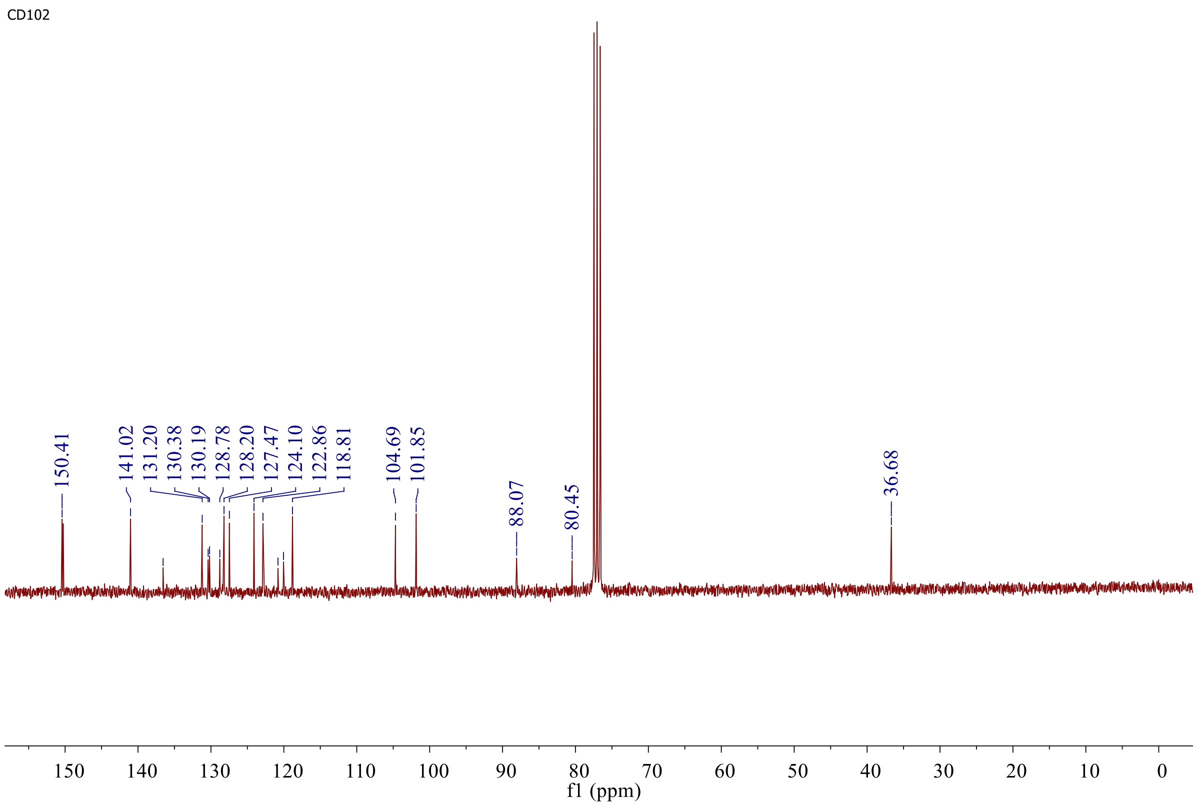

Supplementary Fig. 16: ^{13}C NMR spectrum of isomer (CD102) in $CDCl_3$.

Isomer CD102: ^{1}H NMR (300 MHz, $CDCl_3$): 8.59 (d, *J*= 2.2 Hz,1H); 8.55 (d, *J*= 1.6 Hz, 1H); 8.14 (s, 1H) ; 7.95 (d, *J*= 9.2 Hz, 1H); 7.82 (s, 1H); 7.47-7.28 (m, 2H); 6.69 (d, *J*= 3.4 Hz,1H); 5.19 (s, 2H).^{13}C NMR (300 MHz, $CDCl_3$): 150.41, 141.02, 136.56, 131.20, 130.38, 130.19, 128.78, 128.20, 127.47, 124.10, 122.86, 118.81, 104.69, 101.85, 88.07, 80.45, 36.68.
